# Expression of ChAT, Iba-1, and nNOS in the Central Nervous System following Facial Nerve Injury

**DOI:** 10.3390/antiox13050595

**Published:** 2024-05-12

**Authors:** Jae Min Lee, Myung Chul Yoo, Yong Jun Kim, Sung Soo Kim, Seung Geun Yeo

**Affiliations:** 1 Department of Otorhinolaryngology, Head & Neck Surgery, College of Medicine, Kyung Hee University, Seoul 02447, Republic of Korea; sunjaesa@hanmail.net; 2 Department of Physical Medicine & Rehabilitation, College of Medicine, Kyung Hee University, Seoul 02447, Republic of Korea; famousir@naver.com; 3 Department of Pathology, College of Medicine, Kyung Hee University, Seoul 02447, Republic of Korea; yjkim1@khu.ac.kr; 4 Department of Biochemistry and Molecular Biology, College of Medicine, Kyung Hee University, Seoul 02447, Republic of Korea; sgskim@khu.ac.kr

**Keywords:** facial nerve injury, ChAT, Iba-1, nNOS, central nervous system

## Abstract

Facial nerve injury can cause significant functional impairment, impacting both the peripheral and central nervous systems. The present study evaluated changes in facial motor function, numbers of cholinergic neurons and microglia, and nNOS levels in the facial nucleus of the central nervous system (CNS) following peripheral facial nerve injury. Facial nerve function, as determined by eyeblink and whisker-movement reflexes, was evaluated at baseline and 1, 2, 3, 4, 8, and 12 weeks after inducing facial nerve injury through compression or axotomy. The expression of choline acetyltransferase (ChAT), ionized calcium-binding adaptor molecule 1 (Iba-1), and neuronal nitric oxide synthase (nNOS) in the facial nucleus of the CNS was analyzed 2, 4, and 12 weeks after peripheral facial nerve injury. Compression-induced facial nerve injury was found to lead to temporary facial motor impairment, whereas axotomy resulted in persistent impairment. Moreover, both compression and axotomy reduced ChAT expression and increased Iba-1 and nNOS expression in the facial nucleus, indicating upregulation of an inflammatory response and neurodegeneration. These results indicate that, compared with compression-induced injury, axotomy-induced facial nerve injury results in greater facial motor dysfunction and more persistent microglial and nitric oxide activation in the facial nucleus of the CNS.

## 1. Introduction

The facial nerve (CN VII), vital for facial expressions, taste and salivary gland function, is prone to damage, resulting in symptoms such as facial paralysis, loss of taste, and dry mouth [1]. Peripheral facial nerve paralysis, in turn, can lead to drooping brows, incomplete eyelid closure, dry eyes, hyperacusis, and difficulty closing the mouth [2]. In addition to directly affecting peripheral nerves, peripheral facial nerve injury induces changes in the central nervous system (CNS) through various mechanisms [3]. Following peripheral nerve injury in a rat model, motor neurons in the brainstem undergo damage, leading to neuronal injury characterized by cell membrane damage, inflammatory response, and cell death [4,5]. This damage affects various parts of the nervous system, including nerve cells involved in acetylcholine synthesis and secretion [6].

Cholinergic neurons primarily synthesize acetylcholine, which is produced in the cytoplasm by choline acetyltransferase (ChAT) [7]. These cholinergic neurons are primarily linked to motor neurons, which transmit motor signals to the neuromuscular junction to activate muscles. Therefore, by synthesizing and transmitting acetylcholine, cholinergic neurons play a crucial role in regulating the activity of motor neurons involved in muscle contraction. Cholinergic neurotransmitters in the facial nucleus of the CNS are downregulated after axotomy, disrupting signaling processes between nerves and muscles [8]. Axotomy impairs nerve cell functions involved in muscle movement, hindering proper synthesis and the secretion of acetylcholine. This results in changes in electrical activity or synapse stability that ultimately affect acetylcholine secretion and receptor interaction [6]. These post-axotomy alterations in facial nerve cells may compromise muscle control and CNS function, leading to facial paralysis, muscle weakness, and other neurological symptoms [9]. Axotomy of the rat facial nerve results in decreased motor activity in the ipsilateral nucleus, accompanied by a reduction in the level of ChAT in damaged facial motor neurons [10].

Following peripheral facial nerve injury, inflammatory responses are upregulated in the CNS [11], possibly owing to damaged nerve cells and inflammatory changes in the surrounding tissues [12]. Damaged nerve cells trigger microglial activation and the release of inflammatory signals, leading to an immune response in adjacent tissues. Ionized calcium-binding adapter molecule 1 (Iba-1) serves as a marker for the activity of microglia–immune cells predominantly found in the CNS [13]. This heightened expression of Iba-1 is an indicator an inflammatory response or immune activation within the CNS. After peripheral facial nerve injury, nerve cell damage and microglial responses may increase in the facial nucleus [14]. The inflammatory response induced by axotomy in the CNS can impact neuronal survival and function, thereby playing a pivotal role in neuronal injury and recovery mechanisms [15].

The inflammatory response triggered by peripheral facial nerve injury is correlated with increased CNS production of nitric oxide (NO) reflecting the activation of nitric oxide synthase (NOS) [16]. NOS, the enzyme that produces NO within cells, plays diverse physiological and pathological roles in the CNS and peripheral nervous system. The neuronal form of NOS (nNOS) is involved in neurotransmission and neuronal survival in the CNS. nNOS, primarily expressed in the CNS, plays a crucial role in the production of NO, a key signaling molecule. Several studies have shown that nNOS levels are increased in damaged neurons, where they contribute to neuroprotection and repair mechanisms [17,18]. In particular, peripheral nerve damage caused by facial nerve axotomy induces increased NOS production in motor neurons [19]. Overexpression of nNOS triggered by peripheral nerve injury is ultimately associated with neurodegeneration [20]. In addition to causing neuronal damage by promoting oxidative stress, the excessive NO produced as a result of this overexpression can induce neurodegeneration by triggering inflammatory responses, which further impair neuronal function.

The aims of this study were to assess the expression of ChAT, Iba-1, and nNOS in the CNS following facial nerve injury produced by nerve compression or axotomy and to identify resultant nerve cell damage in the CNS.

## 2. Materials and Methods

### 2.1. Animals

Male Sprague–Dawley (SD) rats weighing 200–250 g at 6 weeks of age were procured from Orient Bio, Seongnam, Gyeonggi-do, Republic of Korea. Rats, housed in a controlled environment (22 ± 2 °C, 50% humidity), were maintained under a 12 h light/dark cycle and provided ad libitum access to food and water. Following a 1-week acclimatization period, rats were randomly assigned to Compression, Axotomy or Sham groups. Rats were sacrificed at 2, 4, and 12 weeks after facial nerve injury; rats in the Sham group received a postauricular incision and were sutured under inhalation anesthesia without facial nerve injury.

### 2.2. Induction of Facial Nerve Injury

Before inducing facial nerve injury (compression or axotomy) in rats, inhalational anesthesia was initiated with 5% isoflurane (Foran solution, Joongwae, Hwaseong, Gyeonggi-do, Republic of Korea) and 80% oxygen and maintained with 3% isoflurane. Under inhalational anesthesia, a left postauricular incision was made to expose the parotid gland and mastoid process. In the Sham-operated group, wounds were sutured after the incision. In all other rats, the facial nerve trunk and its five branches (temporal, zygomatic, buccal, mandibular, and cervical) were identified. Compression was induced by compressing the facial nerve trunk for 30 s using forceps at the midpoint between the area of emergence of the left facial nerve trunk and the area of branching of the facial nerve. Axotomy was induced by completely severing a 5 mm segment of the facial nerve. After manipulations, the incised skin wounds were sutured, and the rats were allowed to recover from anesthesia. Weight, behavior, and wound problems were monitored for 3 days after surgery.

### 2.3. Behavioral Tests

Following facial nerve injury, the extent of damage and recovery was assessed at 1, 2, 3, 4, 8, and 12 weeks by monitoring whisker movement by the vibrissae muscle and the blink reflex of the eyelid. The vibrissae movement test, employed to measure whisker movement by the vibrissae muscle, involved securely holding the rat with both hands and evaluating the extent of whisker movement using the five-point vibrissae observation scale. A score of 5 points, defined as normal, was assigned when whiskers on the injured side moved to the front position similarly to those on the uninjured side. A score of 4 points was given for normal movement but tilted backward, 3 points for significant movement with backward tilt, 2 points for slight movement with the whiskers laid back, and 1 point for no movement with the whiskers laid back.

The eye closure, blinking reflex test was utilized to measure the blink reflex of the eyelid. This involved stimulating the area around the eyes with air puffs of equivalent intensity using an air pump. The degree of eyelid closure was evaluated using the eye closing and blinking reflex observation scale. A score of 5 points was assigned for complete closure, 4 points for 75% closure, 3 points for 50% closure, 2 points for contraction without closure, and 1 point for no movement of the eyelid.

### 2.4. Tissue Preparation

Rats were anesthetized with ether at 2, 4, and 12 weeks after facial nerve injury, followed by perfusion with 0.01 M phosphate-buffered saline (PBS) and exposure of their brains. For immunohistochemical analyses, the brains were subsequently perfused with 4% paraformaldehyde (PFA), removed and fixed overnight at 4 °C in 4% PFA. The brains were dehydrated by immersion in a 30% sucrose solution at 4 °C for 3 days, and serially sectioned into consecutive 40 μm coronal slices using a cryostat maintained at a low temperature. The brain sections were stored in a solution composed of 30% ethylene glycol, 30% glycerin, 0.5 M phosphate buffer (PB), pH 7.4, and 36% distilled water.

### 2.5. Immunohistochemistry

The levels of expression of ChAT, Iba-1, and nNOS were determined immunohistochemically. Antigen retrieval was performed by immersing tissue sections in citrate buffer (pH 6.0) for 20 min in a steamer. Following blocking with 10% normal goat and rabbit serum for 1 h, an average of three brain sections from each region were selected, mounted onto gelatin-coated slides, and incubated overnight with anti-ChAT (1:1000; Merck Millipore, Billerica, MA, USA), anti-Iba-1 (1:500; Abcam, Cambridge, UK), and anti-nNOS (1:500; Cell Signaling Technology, Danvers, MA, USA) antibodies diluted in 3% bovine serum albumin (BSA) and 0.3% Triton X-100. The sections were washed three times for 5 min each with PBS and incubated with rabbit or goat secondary antibodies (1:200; Vector Laboratories, Burlingame, CA, USA) for 1 h at room temperature. The sections were washed three times with PBS for 5 min each and incubated for 1 h in 0.3% Triton X-100. After air drying overnight at room temperature, the sections were covered with curved glass coverslips and mounted using Permount (Vector Laboratories, Burlingame, CA, USA).

### 2.6. Statistical Analysis

The data presented herein represent the means of at least two replicates and are expressed as means ± standard error of the mean (SEM). Statistical analyses were conducted using the SPSS statistical package, version 25 (IBM SPSS Corp., Armonk, NY, USA). Experimental results were compared using one-way analysis of variance (ANOVA), followed by post hoc Tukey tests for group comparisons. A significance level of *p* < 0.05 was established to indicate statistically significant differences.

## 3. Results

### 3.1. Eyelid Blink Reflex and Whisker Movement Tests

**Figure 1:**
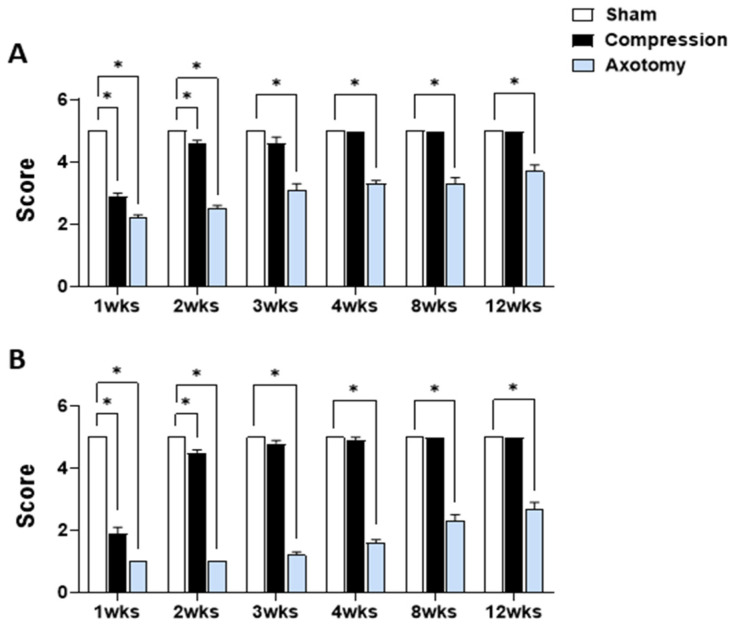
(**A**) Eyelid blink reflex and (**B**) whisker movement (vibrissae muscle) tests at 1, 2, 3, 4, 8, and 12 weeks after facial nerve injury induced by compression or axotomy. Data are expressed as means ± S.E.M. (* *p* < 0.05 vs. Sham group).

Figure 1 displays the outcomes of eyelid blink reflex tests and tests of whisker movement by the vibrissae muscle conducted 1, 2, 3, 4, 8, and 12 weeks after facial nerve injury produced by compression or axotomy. Scores of the eyelid blink reflex test (Figure 1A) were significantly lower in Compression and Axotomy groups compared with the Sham group at 1 (F = 217.175, *p* < 0.001) and 2 (F = 149.203, *p* < 0.001) weeks after facial nerve injury. At 3 weeks post-facial nerve injury, no significant difference was observed between the Compression group and the Sham group, indicating recovery of facial nerve function following compression. In contrast, the Axotomy group consistently exhibited significantly lower scores than the Sham group at 3 (F = 54.534, *p* < 0.001), 4 (F = 137.5, *p* < 0.001), 8 (F = 62.5, *p* < 0.001), and 12 (F = 40, *p* < 0.001) weeks after facial nerve injury.

In the whisker movement (vibrissae muscle) test (Figure 1B), whisker movement scores were significantly lower in both Compression and Axotomy groups compared with the Sham group at 1 (F = 520.686, *p* < 0.001) and 2 (F = 969, *p* < 0.001) weeks after facial nerve injury. Similar to results of eyeblink tests, no significant difference in whisker movement was observed between the Compression group and Sham group 3 weeks after facial nerve injury (*p* > 0.05), indicating recovery of facial nerve function following compression. In contrast, the Axotomy group continued to display significantly lower scores than the Sham group, even at 3 (F = 597.032, *p* < 0.001), 4 (F = 392.413, *p* < 0.001), 8 (F = 160, *p* < 0.001), and 12 (F = 122.5, *p* < 0.001) weeks after facial nerve injury.

### 3.2. Expression of ChAT in the Facial Nucleus following Facial Nerve Injury

**Figure 2:**
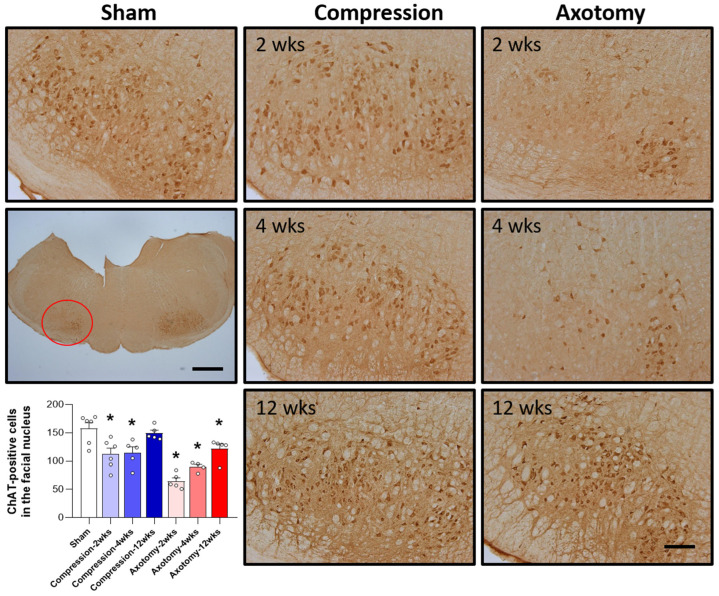
Immunohistochemical analysis of changes in ChAT-positive cells in the facial nucleus following compression- or axotomy-induced facial nerve injury. Representative images of immunostained sections from Sham, Compression, and Axotomy groups show ChAT-positive cells in the facial nucleus at 2, 4, and 12 weeks after facial nerve injury. Scale bar: 200 μm. Bottom left: Summary data quantifying immunostaining results. Data are expressed as means ± S.E.M. (* *p* < 0.05 vs. Sham group).

The expression of ChAT, the main enzyme that synthesizes the important neurotransmitter acetylcholine, was assessed in the facial nucleus 2, 4, and 12 weeks after facial nerve injury induced by compression and axotomy. Compared with Sham-operated rats, compression significantly reduced the number of ChAT-positive cells in the facial nucleus at 2 and 4 weeks (F = 6.988, *p* < 0.01), but not at 12 weeks, indicating that, following an initial reduction in response to a compression-induced facial nerve injury, facial nerves recover at 12 weeks. In contrast, axotomy significantly reduced the number of ChAT-positive cells compared with Sham-operated rats at 2, 4, and 12 weeks (F = 26.861, *p* < 0.001) (Figure 2), suggesting that the reduction in ChAT-positive cells following facial nerve axotomy is sustained.

### 3.3. Expression of Iba-1 in the Facial Nucleus following Facial Nerve Injury

**Figure 3:**
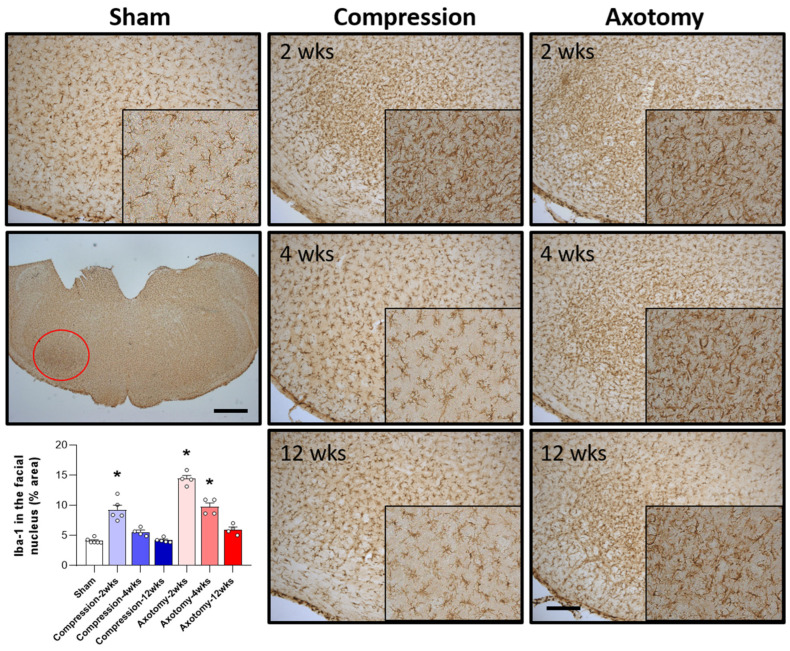
Immunohistochemical analysis of changes in Iba-1 in the facial nucleus following compression- or axotomy-induced facial nerve injury. Representative images of immunostained sections from Sham, Compression, and Axotomy groups show Iba-1 expression in the facial nucleus at 2, 4, and 12 weeks after facial nerve injury. Scale bar: 200 μm. Bottom left: Summary data quantifying immunostaining results. Data are expressed as means ± S.E.M. (* *p* < 0.05 vs. Sham group).

The numbers of microglial cells in the facial nucleus 2, 4, and 12 weeks after facial nerve injury were assessed by measuring the levels of Iba-1, a marker for hyperactive microglial cells. Compared with Sham-operated rats, both Compression and Axotomy significantly increased the number of Iba-1-positive cells at 2 weeks (F = 31.122, *p* < 0.001), suggesting that microglial cells became hyperactive in response to the initial facial nerve injury. At 4 and 12 weeks, however, Iba-1 expression did not differ between the Compression and Sham-operated rats, suggesting that the resolution of microglial hyperactivity following compression-induced injury was relatively rapid. In contrast, Iba-1 expression remained significantly higher in axotomy than in Sham-operated rats at 4 weeks (F = 87.9, *p* < 0.001), although there was no significant difference at 12 weeks (Figure 3). These findings suggested a less rapid decrease in microglial cell activity following facial nerve axotomy than compression, potentially indicating that temporal responses differ following axotomy- and compression-induced injuries.

### 3.4. Expression of nNOS in the Facial Nucleus following Facial Nerve Injury

**Figure 4:**
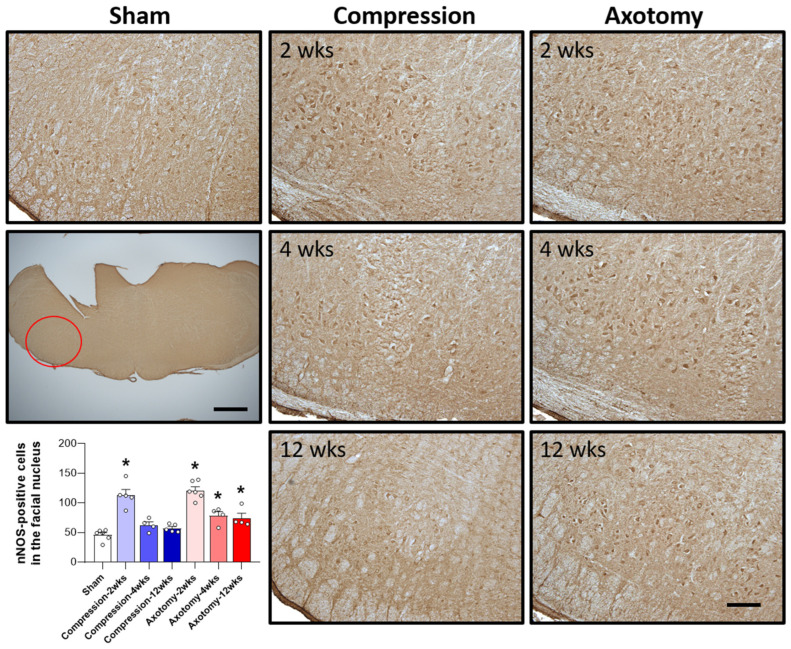
Immunohistochemical analysis of changes in nNOS in the facial nucleus following compression- or axotomy-induced facial nerve injury. Representative images of immunostained sections from Sham, Compression, and Axotomy groups show eNOS expression in the facial nucleus at 2, 4, and 12 weeks after facial nerve injury. Scale bar: 200 μm. Bottom left: Summary data quantifying immunostaining results. Data are expressed as means ± S.E.M. (* *p* < 0.05 vs. Sham group).

The expression of nNOS, an enzyme predominantly expressed in neurons that plays a crucial role in NO generation, was also assessed 2, 4, and 12 weeks after facial nerve injury. At 2 weeks the expression of nNOS was significantly higher in rats that underwent compression or axotomy than in Sham-operated rats (F = 33.247, *p* < 0.001), suggesting an initial physiological response to facial nerve injury that leads to elevated nNOS expression. At 4 weeks, there was no significant difference in nNOS expression between the Compression and Sham-operated groups, indicating that nNOS expression had returned to baseline levels 4 weeks after compression-induced facial nerve injury. In contrast, the initial increase in nNOS expression following axotomy-induced facial nerve injury was sustained at 4 and 12 weeks, indicating continuous generation of NO (F = 39.384, *p* < 0.001). Taken together, these findings indicate that compression-induced facial nerve injury results in an initial increase in nNOS expression that normalizes after 4 weeks, whereas axotomy-induced damage leads to a persistent elevation in nNOS expression, suggesting that this type of facial nerve injury results in prolonged NO production and potential long-term consequences (Figure 4).

## 4. Discussion

In this study, we induced facial nerve injury through compression or axotomy of the peripheral facial nerve and confirmed damage by assessing eyeblink and whisker-movement reflexes. We found that compression-induced peripheral facial nerve injury resulted in a temporary reduction in both eyeblink reflex and whisker movement reflex compared with the Sham group. These effects lasted up to 2 weeks, but facial nerve function showed signs of recovery after 3 weeks. In contrast, peripheral facial nerve injury caused by axotomy led to a sustained impairment in facial nerve function, as evidenced by continued deficits in the eyeblink reflex and whisker movement reflex, even after 12 weeks. This highlights the severe and prolonged impairment of facial nerve function following axotomy-induced peripheral facial nerve injury. This finding is consistent with a previous report that facial nerve function, assessed by measuring eyeblink reflex and whisker movement reflex, did not recover even 12 weeks after facial nerve axotomy [21].

Our study further showed that ChAT expression levels were decreased in the facial nucleus 2 and 4 weeks after facial nerve compression but recovered by 12 weeks. However, ChAT expression levels remained reduced at all time points tested (2, 4, and 12 weeks) after facial nerve axotomy. ChAT, primarily produced within the facial nucleus of the CNS, serves as a pivotal enzyme in acetylcholine synthesis. Hence, the decrease in ChAT levels could potentially influence motor function. Whereas ChAT expression in the Compression group showed recovery by 12 weeks, the Axotomy group exhibited a gradual trend toward recovery, albeit without reaching baseline levels. In another peripheral nerve injury model—hypoglossal nerve axotomy in adult rats—ChAT and vesicular acetylcholine transporter (VAChT) mRNA expression were decreased in the hypoglossal nucleus 4 weeks after injury [22]. Acetylcholine, synthesized in the cytoplasm of cholinergic neurons by ChAT, is packaged into synaptic vesicles by VAChT. Interestingly, the VAChT gene is located within the first intron of the ChAT gene, an arrangement that suggests a tight regulatory relationship between the two components essential for acetylcholine production and transport. The observed decrease in the expression of ChAT and VAChT in the hypoglossal nucleus after transection of the peripheral hypoglossal nerve suggests that transcription of cholinergic genes is closely linked to motor neuron activity. A previous study reported that ChAT levels were downregulated in the facial nucleus 2 weeks after peripheral facial nerve transection without accompanying damage to facial motor neurons [23]. Furthermore, in a mouse model of peripheral facial nerve injury, ChAT levels were reported to remain downregulated in the facial nucleus, even 4 weeks after facial nerve axotomy [24]. These results suggest that peripheral facial nerve injury reduces ChAT levels in the facial nucleus of the CNS, and that the reduction in ChAT is associated with motor dysfunction.

Our results showed hyperactivity of microglial cells in the facial nucleus after facial nerve injury following compression- or axotomy-induced injury. Consistent with this, a previous report showed the activation of a microglial response in the facial nucleus after axotomy-induced facial nerve injury [9]. Similarly, sciatic injury caused by compression or total transection was shown to be accompanied by activation of microglial cells in dorsal and ventral spinal cord horns [25]. Moreover, microglial activation was reported in the dorsal horn of the spinal cord as well as the ventral horn of the spinal cord around the cell bodies of damaged motor neurons ipsilateral to the injury after unilateral sciatic nerve transection [26]. In addition, peripheral nerve damage induced by chronic constriction injury of the sciatic nerve was shown to result in activation of Iba-1 and glial fibrillary acidic protein in dorsal root ganglia of the CNS [27]. The activation of microglia and astrocytes in the dorsal root ganglia is a common response to peripheral nerve injury and is associated with neurodegeneration. The activation of microglia cells in the CNS releases pro-inflammatory cytokines, chemokines and neurotrophic factors, leading to neuronal damage. This process is associated with increased inflammatory responses in the CNS. Moreover, it has been shown that inflammatory factors increase in the CNS after peripheral nerve injury. These results suggest that increased inflammatory responses in the CNS following facial nerve injury lead to the activation of microglia.

Our study demonstrated that both peripheral facial nerve compression and axotomy resulted in upregulation of nNOS expression within the facial nucleus. Under normal physiological conditions, NO exerts anti-inflammatory effects, but its overproduction in abnormal circumstances can lead to inflammation and contribute to apoptotic responses. Facial nerve damage can trigger nNOS activation, which subsequently regulates inflammatory responses in surrounding tissues. A previous study showed an increase in nNOS expression in motor neurons of the inferior brainstem following peripheral nerve compression in Wistar rats [20]. It has also been shown that nNOS expression is increased in dorsal root ganglia of the CNS after chronic constriction injury of the sciatic nerve [27] and in the hypoglossal nucleus 14 days after peripheral hypoglossal nerve transection [28]. Previous research has demonstrated that severing the peripheral facial nerve and administering a NOS inhibitor facilitates axon regeneration [29], suggesting that NOS inhibitors enhance axon regeneration by modulating nNOS activity. This is supported by findings showing a close correlation between the reduction in motor neurons after axotomy in neonatal mammals and NOS activity [30]. Additionally, suppressing NO and NOS expression following facial nerve transection was shown to improve the survival time of motor neurons in the brainstem [31]; evidence on the subject is conflicting, with one report indicating that the elevation of nNOS levels resulting from peripheral nerve damage contributes to the restoration of facial nerve distribution during nerve regeneration [32]. Moreover, increased nNOS expression with peripheral nerve injury has been reported to be associated with both neuronal damage and recovery processes [33]. Our results showed that nNOS was predominantly increased in the facial nucleus at 14 days after compression- and axotomy-induced facial nerve injury. This increase did not persist beyond 28 days in the Compression group, but was sustained even after 3 months in the Axotomy group. Our results, taken together with several previous studies, suggest that, whereas nNOS is associated with nerve regeneration, the overexpression of nNOS induced by injury may be more closely linked to nerve degeneration. Thus, axotomy-induced peripheral facial nerve injury leads to a long-term increase in nNOS expression that is associated with neurodegeneration.

One limitation of our study is the absence of discernible differences between the phenotypes of microglia activated by compression- and axotomy-induced facial nerve injury. The activation of M1 microglia contributes to cytotoxicity and inflammation driven by pro-inflammatory cytokines, whereas the activation of M2 microglia is associated with anti-inflammatory effects. Exploring phenotypic changes in microglial cells within the facial nucleus following peripheral facial nerve injury may provide insights into the relationships between inflammatory responses and nNOS expression during recovery from facial nerve dysfunction.

The results of the present study may have clinical implications for the management of patients with facial nerve damage due to compression and axotomy. Compression may cause temporary damage, but recovery is often possible after a period of time. Axotomy, however, may cause more lasting damage, with recovery being more difficult. These findings may have implications for clinical management or therapeutic interventions. For example, expected recovery times and effects may be dependent on the type of facial nerve injury, making it necessary to accurately assess the type and extent of damage and develop an appropriate treatment plan. Additionally, damage to peripheral facial nerves may induce changes in the central nervous system, which can directly affect the recovery of a patient’s neurological function, improving treatment and rehabilitation programs. Therefore, accurately assessing changes in the central nervous system can play an important role in improving patient treatment and recovery.

## 5. Conclusions

These findings underscore the more enduring and more severe consequences of facial nerve injury resulting from axotomy than from compression. The recovery of ChAT neurons and the concomitant increases in nNOS and Iba-1 expression within the facial nucleus following peripheral nerve injury appear to be interlinked. Damage to ChAT neurons in response to peripheral nerve injury leads to a reduction in acetylcholine levels, initiating an inflammatory cascade that upregulates the expression of nNOS and Iba-1. However, as the recovery of ChAT neurons progresses over time, acetylcholine levels rebound, gradually dampening the inflammatory response and subsequently reducing the levels of expression of nNOS and Iba-1. This suggests that the restoration of ChAT neurons normalizes acetylcholine levels, mitigating the inflammatory response and subsequently reducing the levels of expression of nNOS and Iba-1. These mechanistic insights may have profound implications for both physiological function and pathological outcomes following facial nerve injury.

## Data Availability

Data are contained within the article.
